# An Investigation Into Carbapenem Resistance in Enterobacteriaceae Among Outpatients With Urinary Tract Infection in Southwestern Uganda

**DOI:** 10.7759/cureus.72387

**Published:** 2024-10-25

**Authors:** Barbra Tuhamize, Deusdedit Tusubira, Charles Masembe, Pascal Bessong, Joel Bazira

**Affiliations:** 1 Department of Microbiology and Parasitology, Mbarara University of Science and Technology, Mbarara, UGA; 2 Department of Biochemistry, Mbarara University of Science and Technology, Mbarara, UGA; 3 Department of Zoology, Entomology, and Fisheries Sciences, Makerere University, Kampala, UGA; 4 Department of Microbiology, University of Venda, Thohoyandou, ZAF

**Keywords:** carbapenamases, carbapenem resistance, carbapenems, enterobacteriaceae, urinary tract infection

## Abstract

Background and aim

Urinary tract infections (UTIs) are among the most common bacterial infections worldwide, primarily caused by Enterobacteriaceae. The rise of carbapenem-resistant Enterobacteriaceae (CRE), complicates treatment of UTIs, yet the distribution of CRE and carbapenemase genes in Uganda’s hospitals is not sufficiently explored. This study aimed to examine the distribution of carbapenemase genes in Enterobacteriaceae isolated from urinary tract infections in outpatients in southwestern Uganda.

Methods

A cross-sectional hospital-based study was conducted in southwestern Uganda. The study involved 111 participants who tested positive for carbapenemase genes. These participants were selected from a total of 2,371 patients presenting with urinary tract infections (UTIs) at Bwizibwera Health Center IV and Rubaya Health Center III. Enterobacteriaceaewere identified using a series of biochemical tests, and the presence of carbapenemase resistance genes (blaVIM, blaOXA-48, blaNDM, blaKPC, and blaIMP) was confirmed through polymerase chain reaction (PCR) genotyping. Data were analyzed and presented as frequencies and proportions, displayed in tables and charts.

Results

We screened a total of 2,371 participants with symptoms of urinary tract infections (UTI) for Enterobacteriaceae, 455 (19.2%) tested positive for at least one of the Enterobacteriaceae species. Disk susceptibility testing (DST) for carbapenems (meropenem and ertapenem) revealed a phenotypic carbapenem resistance prevalence of 5.7% (26/455), while polymerase chain reaction (PCR) identified a genotypic prevalence of 24.4% (111/455). *Klebsiella* *spp*. was the most common carbapenemase gene carrier (60/111, 54.1%), with blaIMP being the most frequent gene detected (32.4%). PCR detected more carbapenemase-producing organisms compared to DST. Notably, 14.4% of the isolates harbored multiple carbapenem resistance genes, with one sample carrying four different genes.

Conclusion

Our study revealed a high genotypic prevalence of CRE, especially in *Klebsiella* *spp.* and *Escherichia* *spp.* isolates with a low phenotypic expression. This suggests that relying solely on DST could miss resistant strains, emphasizing the importance of molecular diagnostics like PCR for accurate detection. Carbapenemase inhibitors should be prescribed alongside carbapenem drugs where CREs are suspected, combined with continued surveillance to help manage CRE and reduce their spread in resource-limited settings.

## Introduction

Urinary tract infections (UTIs) are among the most common bacterial infections worldwide, primarily caused by Enterobacteriaceae. The rise of multidrug-resistant (MDR) pathogens, particularly carbapenem-resistant Enterobacteriaceae (CRE) including *Klebsiella*,* E. coli*, *Proteus spp.*, and *Serratia spp*. complicate the treatment of UTIs. CRE are recognized as a high-priority threat by the World Health Organization (WHO) due to their resistance, even to last-resort carbapenem antibiotics [1].

Carbapenem resistance largely results from carbapenemase enzymes, with genes like Verona imipenemase (blaVIM), imipenemase (blaIMP), oxacillinase-48 (blaOXA-48), New Delhi (blaNDM), and *Klebsiella pneumoniae* carbapenemase (blaKPC) driving the resistance. These genes are often located on mobile genetic elements, facilitating the spread of resistance, notably in healthcare settings [2]. CRE infections extend beyond hospitals, with patients serving as reservoirs and transmitting resistant strains into communities. This is particularly concerning in low-resource settings like Uganda, where inadequate infrastructure and poor sanitation exacerbate the issue [3]. Limited data on CRE prevalence in Uganda’s hospitals raises concerns about healthcare facilities as potential sources of resistance. Discharged patients may unknowingly spread CRE to others, highlighting the need for research into the genetic makeup of carbapenemase-producing Enterobacteriaceae from UTI patients.

In Uganda, the threat posed by antibiotic resistance is growing, exacerbated by factors such as the improper use of antibiotics in both human and veterinary medicine and a lack of robust antimicrobial resistance (AMR) monitoring systems [4]. Understanding the distribution of key resistance genes in southwestern Uganda can inform strategies to curb the spread of CRE. A One Health approach, considering human, animal, and environmental factors, is critical to tackling this growing threat [5].

## Materials and methods

A cross-sectional hospital-based study was conducted in southwestern Uganda, involving 111 participants who tested positive for Enterobacteriaceae harboring carbapenemase genes. These participants were selected from a total of 2,371 outpatients presenting with urinary tract infections (UTIs) at Bwizibwera Health Center IV and Rubaya Health Center III. Enterobacteriaceae isolated from urine samples were identified using a series of biochemical tests including oxidase, citrate utilization, indole, urease production, triple-sugar iron, and motility tests [6]. For susceptibility testing, we used the Kirby-Bauer diffusion method following the Clinical Laboratory Standards Institute (CSLI) guidelines 2019, as described previously [7].

The presence of carbapenemase resistance genes (blaVIM, blaOXA-48, blaNDM, blaKPC, and blaIMP) was confirmed through polymerase chain reaction (PCR) genotyping. To extract DNA, colonies were washed with 1X TE buffer twice. Then, 100 µL TE elution buffer was added to the pellet and vortexed for 1 min. The mixture was incubated at 95°C for 1 hour and 30 min using a heating dry bath and centrifuged at 15,000 rpm for 5 min. The supernatant containing the DNA was transferred to a new ultracentrifuge tube, and the DNA was stored at -20°C until usage within 4 hours [8].

We purchased the PCR kit for gene amplification from New England Biolabs Inc. (M0258L Deep Vent DNA Polymerase, ThermoPol reaction buffer, and magnesium sulfate (MgSO_4_) solution. Each of the open reading frames of specific targets was amplified separately for each sample using forward and reverse primers each with a unique sequence as follows: blaKPC forward primer - TCGTCGCGGAACCATTC, reverse primer - ACAGTGGGAAGCGCTCCTC; blaIMP forward primer - CATGGTTTGGTGGTTCTTGT, reverse primer - ATAATTTGGCGGACTTTGGC; blaVIM forward primer - GATGGTGTTTGGTCGCATA, reverse primer - CGAATGCGCAGCACCAG; blaOXA-48 forward primer - GATTTGCTCCGTGGCCGAAA, reverse primer - CCTTGATCGCCCTCGATT; blaNDM forward primer - CCAATATTATGCACCCGGTCG, reverse primer - ATGCGGGCCGTATGAGTGATTG.

Initial denaturation was at 95°C for 30 s, followed by elongation at 72°C for 1 min, and a final extension at 72°C for 5 min. DNA amplicon was electrophoresed using 1.5% agarose gel, in 1x Tris-Borate EDTA buffer (TBE), 5 µL DSView Nucleic acid stain (cat. no.: M7011), 6X loading buffer (GDSBio Lot 050), and DNA ladder/marker 100 bp (GDSBio Lot 076). Electrophoresis was run at 200V and 80 mA for 1 hour. Bands were visualized using the gene-flash transilluminator. Data were analyzed and presented as frequencies and proportions, displayed in tables and charts. We used KPC-positive control *K. pneumoniae* ATCC BAA-1705, NDM-positive control *K. pneumoniae *ATCC BAA-2146, and a multidrug-resistant strain of *E. coli *harboring multiple carbapenemase genes (blaIMP, blaVIM and blaOXA-48). Distilled water was used as a negative control.

## Results

Study profile

**Figure 1:**
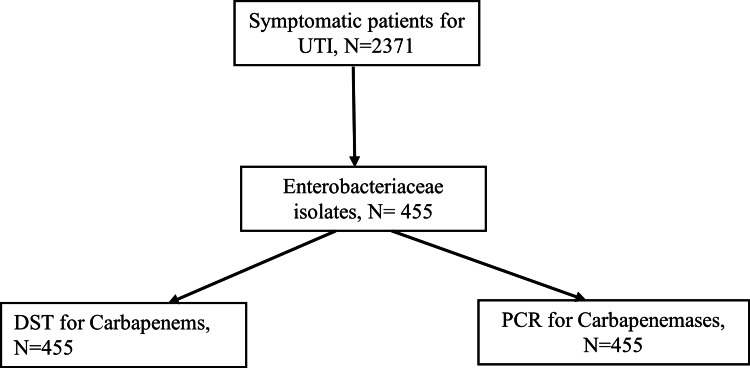
Study flowchart: UTI participants with Enterobacteriaceae. DST: disk susceptibility test; PCR: polymerase chain reaction; UTI: urinary tract infection

A total of 2,371 participants with symptoms of urinary tract infections (UTI) were screened for Enterobacteriaceae, and 455 participants had at least one of the causative organisms identified as Enterobacteriaceae. The identified Enterobacteriaceae were subjected to disk susceptibility testing (DST) using the Kirby-Bauer diffusion method for carbapenem (meropenem and ertapenem) sensitivity and polymerase chain reaction (PCR) for the presence of the carbapenemase genes (Figure 1).

Characteristics and overview of the resistance patterns to carbapenems among study participants

**Figure 2:**
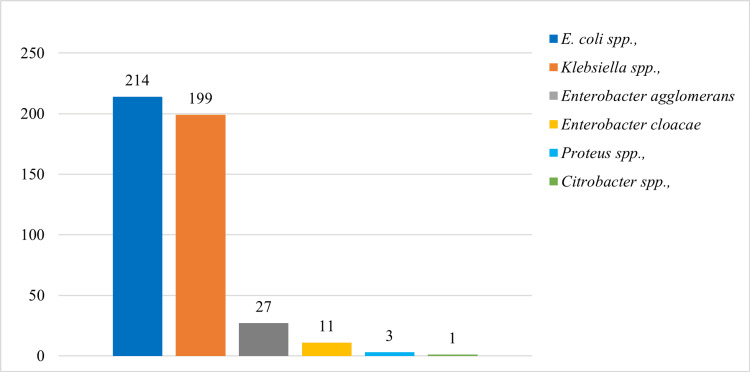
A bar chart showing the Enterobacteriaceae isolated from the study participants. *Escherichia* and *Klebsiella* *spp.* were the predominant organisms.

Out of the screened 2,371 participants, 455 (19.2%) were positive for Enterobacteriaceae, with the majority of the organisms being *Escherichia *and *Klebsiella spp. *(Figure 2). The DST-based phenotypic prevalence of carbapenem resistance was 26/455 (5.7%, 95% CI: 3.8-8.3).

**Figure 3:**
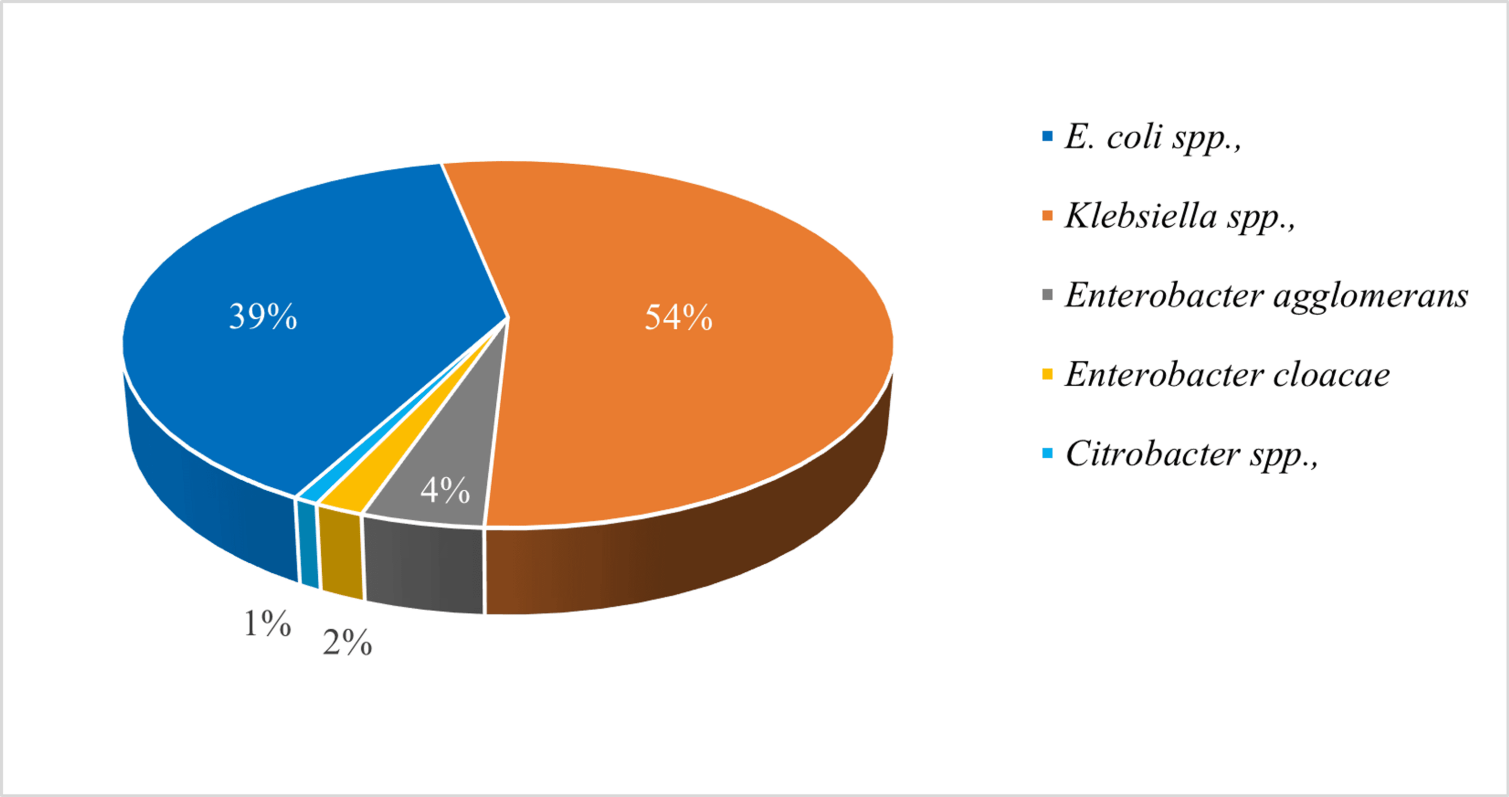
Distribution of carbapenemase genes in Enterobacteriaceae. *Klebsiella* *spp.* had the highest number of organisms that harbored carbapenemase genes as identified by PCR method. PCR: polymerase chain reaction

The genotypic prevalence with PCR was 111/455 (24.4%, 95% CI: 20.5-28.6), with more than half of the organisms harboring a carbapenemase gene being *Klebsiella spp.* (60/111, 54.1%) (Figure 3). PCR method identified more carbapenemase-encoding genes harboring organisms than the DST method with the majority of the genes identified in *Klebsiella *and* Escherichia spp.* (Table 1).

**Table 1:** DST- and PCR-based carbapenem resistance DST: disk susceptibility testing; PCR: polymerase chain reaction

Organism isolated	Resistance pattern	
	DST-based	PCR-based
*Escherichia coli spp*.	7	43
*Klebsiella spp*.	17	60
Enterobacter cloacae	1	2
Enterobacter agglomerans	1	5
Citrobacter spp.	0	1
Proteus spp.	0	0

In general, the carbapenemase genes were disproportionately distributed among the different isolates. blaIMP was the most prevalent carbapenemase-encoding gene (36/111, 32.4%), while blaVIM and blaOXA-48 had similar distribution (23/111, 20.7%) (Table 2).

**Table 2:** The distribution of carbapenemase encoding genes in the study isolates.

Species	Carbapenemase-encoding genes				
	blaIMP	blaNDM	blaKPC	blaVIM	blaOXA-48
Escherichia coli spp.	17	6	12	5	9
Klebsiella spp.	15	17	12	17	10
Enterobacter cloacae	1	0	0	0	1
Enterobacter agglomerans	3	2	0	0	2
Citrobacter spp.	0	0	0	0	1
Proteus spp.	0	0	0	0	0
Total	36	25	24	23	23

In addition, *Proteus spp.* showed neither phenotypic expression nor harbored any resistant gene (Tables 1, 2). The proportion of isolates that harbored more than one resistance gene was 16/111 (14.4%), with blaNDM being the most shared carbapenemase gene. Multiple genes were only carried in *Klebsiella* *spp.*, *Escherichia coli spp.,* and *Enterobacter agglomerans*. Interestingly, one *Klebsiella* *spp.* harbored four out of the five studied genes (Table 3).

**Table 3:** Bacterial isolates positive for carbapenemase genes and number of genes per organism.

Species	Number of isolates	Genes per isolate	bla genes
*Klebsiella spp*.	1	4	VIM, NDM, IMP, and KPC
Klebsiella spp.	1	3	VIM, NDM, and IMP
Escherichia coli spp.	1	3	VIM, NDM, and KPC
Escherichia coli spp.	1	2	KPC and NDM
Escherichia coli spp.	1	2	VIM and NDM
Klebsiella spp.	3	2	VIM and NDM
Klebsiella spp.	1	2	NDM and XA-48
Enterobacter agglomerans	1	2	NDM and OXA-48
Escherichia coli spp.	2	2	IMP and KPC
Escherichia coli spp.	1	2	IMP and OXA-48
Escherichia coli spp.	1	2	NDM and IMP
Klebsiella spp.	2	2	NDM and IMP
Escherichia coli spp.	36	1	IMP or VIM or NDM or KPC or OXA-48
Klebsiella spp.	52	1	IMP or VIM or NDM or KPC or OXA-48
Enterobacter agglomerans	5	1	IMP or NDM or OXA-48
Enterobacter cloacae	2	1	IMP or OXA-48
Citrobacter spp.	1	1	OXA-48
Proteus spp.	3	0	None

## Discussion

Out of the total 2,371 participants screened, 455 (19.1%) tested positive for Enterobacteriaceae, with the majority being Escherichia and Klebsiella species. Carbapenem resistance was phenotypically detected in 5.7% of the isolates using the disk susceptibility test (DST), while genotypic analysis using PCR identified carbapenemase genes in 24.4% of the isolates. This discrepancy highlights the higher sensitivity of molecular techniques in detecting carbapenem resistance, as many CREs may not show phenotypic resistance despite harboring resistance genes. Among the carbapenemase genes identified, blaIMP was the most prevalent (32.4%).

Our study revealed *Escherichia* and* Klebsiella spp*. to be the most predominant organisms responsible for causing UTIs. These Enterobacteriaceae have been reported to cause UTIs in outpatient visits with 72.8% of infections caused by *E. coli* in the 338 US health facilities [9] and 87.6% of the UTIs were due to Enterobacteriaceae* *in the Chicago Emergency Department [10]. In addition, in a review of causative organisms of UTI in Spain, Mancuso et al. reported up to 85% of the UTI being caused by Enterobacteriaceae [11]. An earlier study in Uganda reported more than half (59.2%) of UTIs were due to *Klebsiella* and *E. coli spp*. [12]. These organisms have the ability to form biofilms which enable them to adhere to the urogenital mucous membrane [13,14]. Similar to our current study, *Citrobacter* and *Enterobacter spp*. have been reported in a few studies to be uropathogenic [10,15].

A number of studies have continued to report an increased production of carbapenemases hydrolyzing β-lactam antibiotics, including carbapenems, worldwide [2,16,17]. Our study has revealed a phenotypic and genotypic prevalence of carbapenemase resistance of 5.7% and 22.4%, respectively, among uropathogenic Enterobacteriaceae. Our prevalence is comparable to a study conducted earlier in Uganda, which reported a prevalence of 4.92% in hospital isolates [18]. In addition, Tuhamize et al. reported a comparable prevalence of 5% in human community isolates [7]. The similarity in the findings could be due to the fact that the studies are conducted in similar settings and use similar methods to determine the resistance patterns (DST). Another study carried out in Uganda had a contrasting DST-based prevalence of 18.4% [19]. The difference in the study findings could be because many organisms were studied, isolated from several samples, and used automated methods with better sensitivities to study phenotypic expression. In our study, only urine samples were analyzed using the Kirby-Bauer disk diffusion method with lesser sensitivity.

Lower prevalence has been reported in China (1.0%) [20], Germany (0.3%) [21], and a surveillance study in Spain (0.04%) in hospital isolates with larger sample sizes [22]. In the above countries, there are restrictions on the use of antibiotics by only licensed individuals, contrary to the liberal nature of acquiring drugs from pharmacies over the counter even without a prescription in Uganda [23]. The differences in prescription habits could be responsible for the lower resistance prevalences in China, Spain, and Germany.

Our study revealed that blaIMP was the most prevalent (32.4%) carbapenemase-encoding gene in the studied Enterobacteriaceae. These findings are comparable to an earlier study in neighboring Tanzania, where resistant Gram-negative clinical isolates harbored blaIMP as the prevalent carbapenemase-encoding gene [24]. In contrast, an earlier study of clinical isolates in southwestern Uganda, where our study was carried out, reported no* *blaIMP in the isolates [18]. Our study findings highlight an emerging gene pool of carbapenemase genes to include blaIMP, likely from neighboring countries or societies. However, the prevalence of blaIMP reported by Mushi et al. was higher than in our study, an observation that could be due to the fact they studied various sample isolates and many Gram negatives other than Enterobacteriaceae, while our study only analyzed urine samples with a focus on Enterobacteriaceae [24].

blaVIM has been reported to possess the broadest range of substrate hydrolysis when compared with other carbapenemase genes, with the capability to degrade nearly all β-lactams except monobactams [25]. It is worrisome that in our study, the prevalence of blaVIM was high at 20.7% when compared to previous studies in the region, which reported rates of 19.6% and 12.3%, posing a threat to the use of carbapenems as one of the last-resort antibiotic treatments [18,24]. Surprisingly, a recent study in China reported no blaVIM among the CRE strains isolated from adult and pediatric patients, suggesting a possible differential distribution of carbapenemase-encoding genes [26].

Furthermore, the current study detected blaNDM as highly prevalent (22.5%) among study strains. This is a bothersome statistic given that the NDM-1 encoding gene is documented to be found on different large and easily transmissible plasmids at a high frequency to competent bacteria. These plasmids are thought to confer resistance to several antibiotics [2,27]. Moreover, our study revealed blaNDM as the most prevalent carbapenemase-encoding gene in organisms that harbored multiple genes. In addition, the blaNDM reported in earlier related studies was lower, indicating an increase in the NDM gene pool in the CREs [19,24,28]. Notably, 14.4% of the isolates harbored multiple carbapenem resistance genes, with one isolate containing four genes. The presence of multiple carbapenemase genes within a single organism is concerning, as it may contribute to the heightened resistance of these strains to carbapenems and complicate the use of carbapenems for the treatment of bacterial infections.

Routine implementation of molecular diagnostic tools, such as PCR, should be prioritized in healthcare settings for the detection of carbapenemase genes. Clinicians should consider the use of carbapenemase inhibitors, such as avibactam, alongside carbapenem antibiotics when carbapenemase genes are detected, even in instances where phenotypic resistance is absent, as an approach to hamper the development of resistance [29].

## Conclusions

Our study revealed a high prevalence of carbapenemase genes among Enterobacteriaceae isolated from outpatients with UTIs.* Klebsiella spp.* and *Escherichia spp.* were the most predominant organisms, and blaIMP was the most identified carbapenemase gene. The study also revealed the presence of multiple carbapenem resistance genes in the organisms studied. The blaNDM was the most prevalent carbapenemase-encoding gene in organisms that harbored multiple genes. The routine use of molecular techniques to identify carbapenem-resistant Enterobacteriaceae (CRE) and the co-prescription of carbapenems with a carbapenemase inhibitor require careful consideration.
